# Internal Resonance in a Vibrating Beam: A Zoo of Nonlinear Resonance Peaks

**DOI:** 10.1371/journal.pone.0162365

**Published:** 2016-09-20

**Authors:** Franco Mangussi, Damián H. Zanette

**Affiliations:** Centro Atómico Bariloche and Instituto Balseiro, Comisión Nacional de Energía Atómica, Consejo Nacional de Investigaciones Científicas y Técnicas, 8400 San Carlos de Bariloche, Río Negro, Argentina; Lanzhou University of Technology, CHINA

## Abstract

In oscillating mechanical systems, nonlinearity is responsible for the departure from proportionality between the forces that sustain their motion and the resulting vibration amplitude. Such effect may have both beneficial and harmful effects in a broad class of technological applications, ranging from microelectromechanical devices to edifice structures. The dependence of the oscillation frequency on the amplitude, in particular, jeopardizes the use of nonlinear oscillators in the design of time-keeping electronic components. Nonlinearity, however, can itself counteract this adverse response by triggering a resonant interaction between different oscillation modes, which transfers the excess of energy in the main oscillation to higher harmonics, and thus stabilizes its frequency. In this paper, we examine a model for internal resonance in a vibrating elastic beam clamped at its two ends. In this case, nonlinearity occurs in the form of a restoring force proportional to the cube of the oscillation amplitude, which induces resonance between modes whose frequencies are in a ratio close to 1:3. The model is based on a representation of the resonant modes as two Duffing oscillators, coupled through cubic interactions. Our focus is put on illustrating the diversity of behavior that internal resonance brings about in the dynamical response of the system, depending on the detailed form of the coupling forces. The mathematical treatment of the model is developed at several approximation levels. A qualitative comparison of our results with previous experiments and numerical calculations on elastic beams is outlined.

## Introduction

Vibrating material objects of different kinds and forms have been used for producing and demonstrating oscillatory phenomena since times immemorial. The most ancient musical instruments in the archaeological record, over 40,000 years old, are flutes [1], which produce sound from the vibrations of an air column confined inside a tube. The monochord –a stretched string of controlled length and tension– was widely employed to study the mathematical relations of consonance between tones in traditional Western music theory, presumably, starting from Pythagoras [2]. Elastic deformations of clamped solid beams, which are essential parts of any building structure, were first discussed from a modern scientific perspective around the mid-eighteenth century by Euler and Bernoulli, constituting an early contribution to continuum mechanics [3]. Much more recently, within the field of micro- and nanotechnologies, minute vibrating silica beams clamped at their two ends (clamped-clamped, or c-c beams) have been proposed to replace quartz crystals as pacemakers in the design of time-keeping components (clocks) within miniaturized devices [4–6]. With respect to quartz crystals, the advantages of these micromechanical oscillators range from easiness of fabrication to low power consumption during operation.

The linear theory of elasticity works under the assumption that, when the vibration amplitude is small, the restoring force acting on the beam is proportional to the amplitude itself [7]. Analogously, under the action of an external force, the amplitude is proportional to the force strength. However, linear response breaks down when the beam is driven to vibrate at sufficiently large amplitudes. In this situation, the restoring force on a c-c beam acquires cubic terms, proportional to the third power of the amplitude [8, 9]. The standard model for cubic nonlinearity in mechanical oscillatory motion is given by the Duffing equation, which was first proposed in 1918 by the German engineer Georg Duffing [10], and has been systematically studied as a source of nonlinear phenomena ranging from hysteresis to chaos [11].

In order to overcome the effects of electronic and thermal noise, micromechanical oscillators must vibrate at large amplitudes, thus operating well inside the nonlinear regime [12]. This condition brings about the noxious amplitude-frequency (or a-f) effect [13, 14]: in contrast with a linear oscillator, whose frequency is independent of the amplitude, in its nonlinear counterpart frequency and amplitude are generally interdependent quantities. This means that a variation in the amplitude –due, for instance, to an uncontrolled fluctuation in the driving force– causes a change in the frequency. In a clock, in turn, this change translates into inaccuracy in time keeping.

Another effect of nonlinearity on the dynamics of vibrating beams, which is the main phenomenon addressed in this contribution, is *internal resonance* [15]. As it is well known, an extended elastic object can exhibit different kinds of vibration, called oscillation modes, which differ from each other in their patterns of deformation and frequencies [7]. In a purely linear oscillator, each of these modes can be excited separately –for instance, through the action of an external force– leaving all other modes at rest. Nonlinearity, on the other hand, couples different oscillation modes with each other in such a way that exciting one of them entails energy transfer toward other modes, thus establishing a complex combination of oscillatory patterns. If, moreover, the frequencies of two such modes are mutually syntonized, the corresponding oscillations can synchronize with each other, dramatically enhancing the energy transfer between them, and thus realizing internal resonance. Cubic nonlinearity triggers internal resonance when the frequency ratio between the two modes is close to a multiple of 3. In a recent series of experiments on c-c micromechanical nonlinear oscillators, it has been shown that, if internal resonance is taking place, the oscillation frequency can be drastically stabilized against amplitude variations [16, 17]. As a consequence, the a-f effect referred to in the previous paragraph is neutralized over a wide range of operating conditions. In this situation, remarkably, nonlinear effects of different nature are compensating each other to the advantage of such applications as the design of time-keeping devices.

Here we examine the main mode stationary response near a 1:3 resonance with a higher harmonic mode by means of a model consisting of two coupled oscillators, each of them representing a mode [18]. The 1:3 frequency ratio, in fact, is the primary resonance induced by cubic forces, due to the triple frequency terms appearing when a harmonic function is raised to the third power [19]. The model, which is qualitatively presented at the beginning of the next section and mathematically formulated in the Methods section, allows for a variety of functional forms in the coupling forces between the two modes. Thus, it is able to describe several different responses to internal resonance, likely associated with vibrating beams of different geometries, materials, and design. Moreover, the model can straightforwardly be extended to analyze internal resonance for frequency ratios other than 1:3. Although an exhaustive exploration of the parameters that define coupling is virtually impossible, after studying numerous combinations of parameter values, we have selected a representative set of cases which illustrate the most frequent kinds of behavior. These results, obtained from our mathematical formulation through numerical means, are presented in the main part of next section.

Our presentation focuses on the interrelation between oscillation amplitude, frequency, and phase of the main mode. Experimentally, in fact, these are the most readily accessible quantities characterizing the stationary dynamics of the system [16, 20]. The mathematical formulation developed in the Methods section yields the tools for obtaining the higher harmonic dynamics as well. Moreover, under suitable assumptions, this formulation makes it possible to obtain explicit algebraic expressions that provide an approximate, analytically tractable description of internal resonance. In the concluding section, we summarize our contribution, and point out previous work where resonance phenomena similar to those presented here for the two-oscillator model have been observed in experiments and numerical calculations on actual c-c beams.

## Model and Results

### Two-Oscillator Model for Internal Resonance

In experiments with c-c beam micromechanical oscillators, internal resonance was observed to occur between the main oscillation mode and a higher harmonic mode whose frequency is above three times the frequency of the main mode [16]. The vibration pattern of the main mode is transversal, resembling that of a plucked one-dimensional string. In the higher harmonic vibration, on the other hand, the dimensions perpendicular to the beam length become relevant, and the deformation is torsional. In the experimental setup, the oscillator is excited by a periodic force applied through an electric circuit. If, due to its nonlinear response to the driving force, the frequency of the main mode attains one third of that of the higher harmonic, internal resonance takes place.

To model this behavior, we represent the two oscillation modes as two mutually coupled one-dimensional Duffing oscillators, subject to linear damping and to periodic driving forces (see Methods). The ratio between the natural frequencies of the two oscillators is below 1:3. Coupling between the oscillators is modelled by a generic cubic force –i.e., with the same degree of nonlinearity as the Duffing restoring force– consisting of several contributions proportional to different powers of the two oscillation amplitudes. The cubic nonlinearity is insured if the powers of the two amplitudes in each contribution add up to 3.

When the system attains stationary motion, the two oscillators and the driving force are synchronized in such a way that the frequencies of the main mode oscillator and the force are identical, and exactly equal to one third the frequency of the higher harmonic oscillator. Stationary oscillations are thus characterized by the frequency of the driving force and, additionally, by the amplitudes and phases of the two oscillators. As discussed in the Methods section, these five quantities are linked through four algebraic relations, so that only one of them can be varied independently as a control parameter. In the (open-loop) configuration traditionally considered when studying mechanical nonlinear oscillators, the driving force is applied as an externally controlled action on the system [21]. In this case, the control parameter is the force frequency, which is “copied” by the main mode oscillation and tripled by the higher harmonic mode. On the other hand, in the (closed-loop) self-sustaining configuration used in clock design, the signal produced by the oscillator itself is reinjected as the driving force after shifting its phase and fixing its amplitude [22, 23]. In contrast with the open-loop setup, the parameter controlled by the experimenter in this situation is the phase difference between the force and the main mode, and the stationary oscillation frequency adjusts to this control. While the algebraic relation between frequency, amplitudes, and phases is the same in both configurations, the stability properties of each kind of stationary oscillation may change depending on the parameter being controlled [17].

### Stationary Oscillations without Internal Resonance

**Fig 1:**
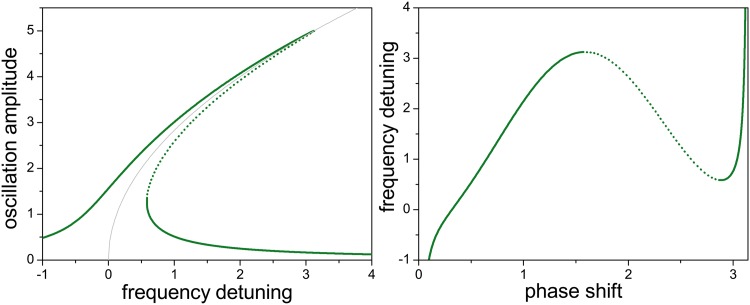
Interdependence between amplitude, frequency, and phase in the absence of internal resonance. Left: Oscillation amplitude vs frequency detuning. Full and dotted lines respectively correspond to stable and unstable oscillations in the open-loop configuration. The light-grey curve stands for the backbone approximation. Right: Frequency detuning vs phase shift. Full and dotted lines correspond to the same stability properties as in the left panel. The units of amplitude and frequency are arbitrary, and the phase shift varies in the interval (0, *π*). The values of the parameters used to obtain the curves are given in Table 1.

Fig 1 illustrates the interdependence of amplitude, frequency, and phase of the main mode oscillator when its coupling with the higher harmonic mode is absent. The left panel shows the stationary oscillation amplitude as a function of the detuning between the driving force frequency and the main mode natural frequency (see Methods for detailed definition of these quantities). This is the well-known “leaning” resonance peak of the forced Duffing oscillator [11], i.e., the counterpart of the “straight” resonance peak of its linear analogue. The case shown in Fig 1 corresponds to a hardening nonlinearity, where the amplitude grows for increasingly positive detuning [15]. This is, in fact, the kind of nonlinearity associated with the main mode oscillation of a c-c beam [8, 9]. The most characteristic feature of the Duffing resonance peak is the existence of a range of frequencies where three amplitudes exist for each frequency. In the open-loop configuration, where the driving force frequency is controlled, the oscillations whose amplitudes are plotted with full and dotted lines are respectively stable and unstable. The light-grey curve shows the so-called backbone of the resonance peak [15], an approximation that –as we show below– helps to estimate the effects of the interaction with the higher mode oscillator in the vicinity of the internal resonance. The formulation of the backbone approximation is discussed in the Methods section.

The right panel of Fig 1 shows the relation between the frequency detuning and the phase shift between the driving force and the main mode, defined as the amount by which the driving force phase precedes the main mode phase. To ease the comparison between the two panels, full and dotted lines in both of them correspond to the same stationary oscillations. To the right, however, we have plotted the phase shift in the horizontal axis as a way of emphasizing its relation with the frequency in the closed-loop configuration, where the control parameter is the phase shift. This representation will become useful when discussing frequency stabilization through internal resonance. In contrast with the open-loop setup, in the closed-loop configuration all the stationary states shown in the figure are stable [22, 23] (below, stability differences in the closed-loop configuration are represented by different colors).

### Internal Resonance with a Linear Higher Harmonic Mode

First, we consider the case where the Duffing cubic nonlinearity in the higher harmonic oscillator is disregarded –namely, we assume that the higher harmonic restoring force is linear. This assumption has been used in the description of internal resonance in micromechanical oscillators [16], and allows for the extension of the backbone approximation referred to above (see Methods). On the other hand, as advanced, coupling between the two oscillators will be described by generic cubic terms.

**Fig 2:**
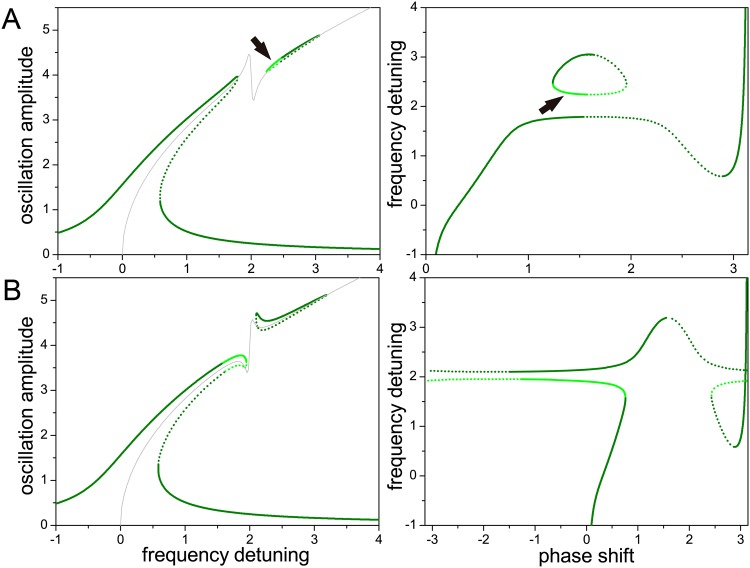
Internal resonance with a linear higher harmonic mode (I). In this case, the coupling force acting on the main mode oscillator is proportional to the higher mode amplitude and to the square of the main mode amplitude. Rows A and B correspond to opposite signs of this coupling force. Full and dotted (respectively, dark- and light-green) lines correspond to stable and unstable oscillations in the open-loop (respectively, closed-loop) configuration. Arrows in row A mark curve segments referred to in the text. The light-grey curve is the backbone approximation. The corresponding parameters are given in Table 1.

In all the cases considered below, the higher harmonic oscillator is subject to a coupling force proportional to the cube of the main mode amplitude. It can be seen from the equations of motion that changing the sign of this force does not affect the oscillation amplitudes, but just adds a fixed amount equal to *π* to the phase of the higher harmonic oscillation. In analogy to Figs 1 and 2 shows the main mode amplitude as a function of the frequency detuning between the driving force and the main mode (left), and the frequency detuning as a function of the phase shift (right), when the main mode oscillator is subject to a coupling force proportional to the higher mode amplitude and to the square of the main mode amplitude. Rows A and B correspond to coupling forces of opposite sign.

As in Fig 1, full and dotted lines in Fig 2 correspond to stable and unstable stationary oscillations in the open-loop configuration. Now, moreover, dark- and light-green curves respectively stand for stable and unstable oscillations in the closed-loop configuration. Note that an unstable oscillation in one of the configurations can be stable in the other, and vice versa. In the left panel of Fig 2A, for instance, the full light-green segment marked with an arrow corresponds to stable (respectively, unstable) oscillations in the open-loop (respectively, closed-loop) configuration. In the right panel, the arrow points to the same oscillations, now in the representation of frequency detuning vs phase shift.

Comparing Figs 1 and 2, we see that a major effect of the interaction between the two oscillators is the appearance of a gap in the main mode resonance peak. The position of the gap coincides with the detuning for which the main mode frequency attains one third of the higher harmonic frequency (see parameters in Table 1), thus realizing internal resonance. Note also that coupling forces of different signs cause contrary deformations of the peak at the two sides of the gap. Outside the gap, these deformations are well described in both cases by the backbone approximation, plotted as a light-grey curve.

**Table 1:** Parameters used in Eq (6) to obtain the results plotted in Figs 1 to 4.

Fig	1	2A	2B	3A	3B	4A	4B
*β*_1_	0	0.5	−0.5	0	0	0	0
*α*_1_	0	0	0	1	−1	1	−1
*α*_3_	0	0	0	0	0	3	−3

The remaining parameters are *ω*_1_ = 1; *ω*_3_ = 9; *σ*_3_ = 6; *μ*_1_ = *μ*_3_ = 0.1; *γ*_1_ = −0.33; *γ*_3_ = 0; *β*_3_ = 0.5; *f*_1_ = 1.

The right panel of Fig 2A illustrates the phenomenon of frequency stabilization induced by internal resonance, which counteracts the a-f effect, as observed in experiments [16, 17, 24]. Indeed, there is a wide interval of phase shifts where the frequency attains a plateau, varying much less than when internal resonance does not occur (cf. right panel of Fig 1). In the closed-loop configuration, where the control parameter is the phase shift, operating within the plateau insures that the system performs stable oscillations with practically constant frequency. Their amplitude lies in the lower part of the resonance peak, at the boundary of the internal resonance gap. For the opposite sign of the coupling force (Fig 2B), meanwhile, two wider parallel plateaus develop –note that, here, the phase shift varies in (−*π*, *π*). In this case, stable oscillations lie in the upper plateau, on the large amplitude “island” of the resonance peak.

**Fig 3:**
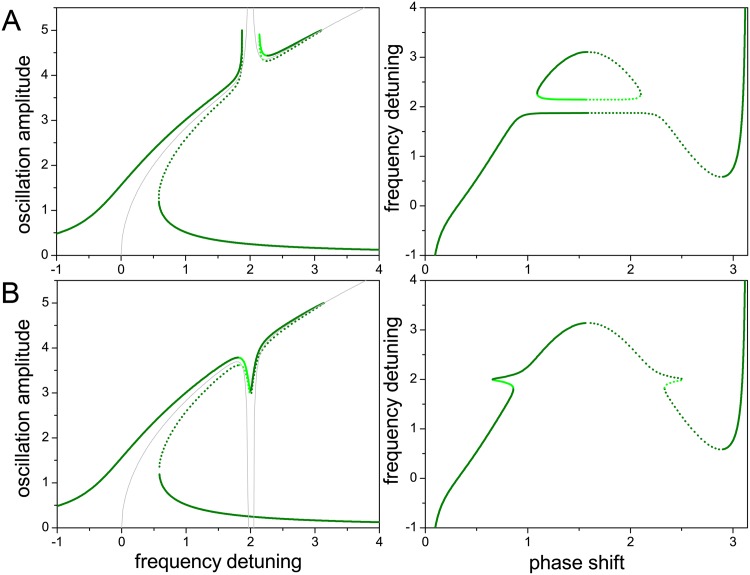
Internal resonance with a linear higher harmonic mode (II). As in Fig 2, when the coupling force acting on the main mode oscillator is proportional to its own amplitude and to the square of the higher harmonic amplitude.

Fig 3 shows internal resonance in the case where the coupling force on the main mode oscillator is proportional to its own amplitude and to the square of the higher harmonic amplitude. Rows A and B correspond to forces of different signs. In the case of Fig 3A, the sharp deformation of the resonance peak toward higher amplitudes at both sides of the internal resonance gap (left panel) is associated with a very flat plateau in the frequency as a function of the phase shift (right panel). In this case, frequency stabilization induced by internal resonance is even more effective that in the case considered in Fig 2A. On the other hand, as shown in Fig 3B, a change in the sign of the coupling force inverts the direction of the deformation and, more remarkably, does not give rise to the internal resonance gap. In both cases, the direction of the deformation is well captured by the backbone approximation.

**Fig 4:**
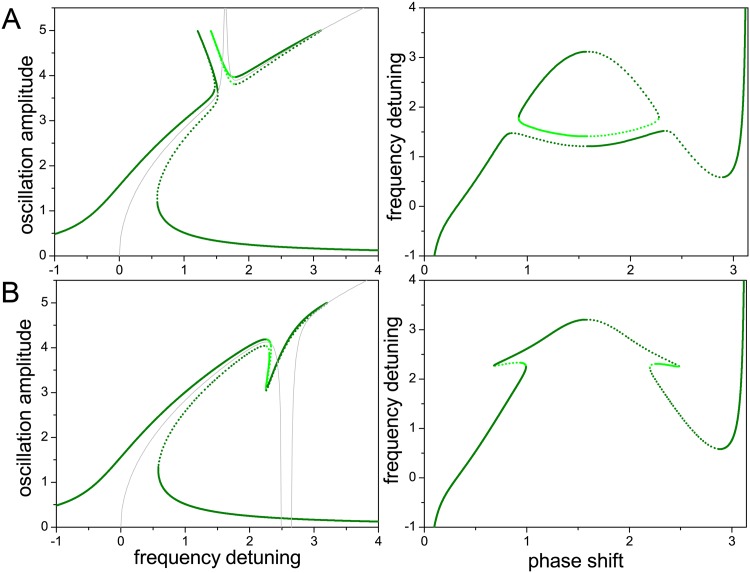
Internal resonance with a linear higher harmonic mode (III). As in Fig 3, with the addition of a coupling force acting on the higher harmonic oscillator. This additional force is proportional to the higher harmonic amplitude and to the square of the main mode amplitude.

A considerable modification to the previous situation occurs if, in addition to the coupling force acting on the main mode oscillator, a force proportional to the higher harmonic amplitude and to the square of the main mode amplitude is applied to the higher harmonic oscillator. Its consequences are illustrated in Fig 4. Comparing with Fig 3 two effects are apparent. First, there is a shift in the frequency detuning at which internal resonance takes place, to smaller values in Fig 4A and larger values in Fig 4B. Second, in both cases, the deformations of the resonance peak bend to the left, towards smaller detuning. Concurrently, the flat plateau in the left panel of Fig 3A becomes distorted, and frequency stabilization is significantly degraded.

The effects of this additional coupling force in the higher harmonic oscillator can be understood taking into account its proportionality to the higher harmonic amplitude itself. Any additional linear force acting on the oscillator will effectively modify its natural frequency –which is defined by the linear restoring force– thus changing the frequency at which the higher harmonic oscillator is able to synchronize with the main mode. The direction of this shift depends on the sign of the force, but its magnitude is also controlled by the main mode amplitude. The shift, therefore, varies along the resonance peak, which explains the bending of the internal resonance deformations. This also explains why frequency stabilization breaks down with this kind of coupling, as the effect of the coupling force is to change the oscillation frequency. From the viewpoint of the dynamical states accessible to the two-oscillator system, it is worthwhile pointing out that the bending of some portions of the resonance peak near the internal resonance creates intervals of frequency detuning where stationary oscillations –both stable and unstable– with up to seven different amplitudes coexist.

**Fig 5:**
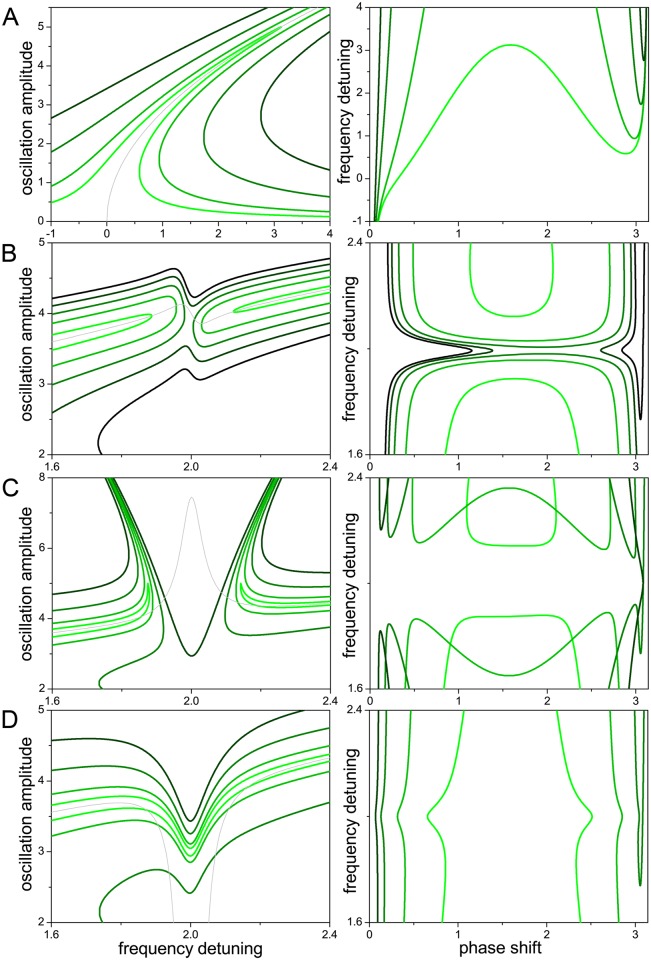
Dependence of internal resonance on the amplitude of the driving force. In all panels, larger force amplitudes are plotted with increasingly darked shades of green (see parameters in Table 2), and differences in stability are disregarded. (A) Effect of increasing the force on the resonance peak in the absence of internal resonance. (B,C,D) Effect on the internal resonance gap for the cases considered in Figs 2A, 3A and 3B, respectively.

The results presented in Figs 1 to 4 have been obtained for a fixed value of the driving force amplitude. Fig 5 illustrates the effect of changing this parameter. In Fig 5A, we see that, in the absence of internal resonance, progressively larger driving strengths (light- to dark-green curves) widen the resonance peak and shift its highest point to larger frequency detuning. Note that the backbone approximation is the same for all curves, as it does not depend on the driving force amplitude (see Methods). Fig 5B and 5C show close-ups in the zone of the internal resonance gap, when increasing the driving force in the cases considered in Figs 2A and 3A, respectively. In both of them, the resulting effect is the closure of the gap when the force attains a sufficiently large value. The driving force is thus effectively competing against the coupling forces, responsible for internal resonance. As expected from the results shown in Fig 5A, the closure of the gap is combined with the widening of the resonance peak. This widening is also apparent in Fig 5D, which corresponds to the case depicted in Fig 3B.

### Internal Resonance with a Nonlinear Higher Harmonic Mode

Experimental results with c-c micromechanical oscillators do not provide evidence that nonlinear behavior in the higher harmonic mode plays any crucial role in the occurrence of internal resonance, but they do show that cubic nonlinearity is in fact present in the higher harmonic dynamics [16]. In experiments, moreover, it appears that the higher harmonic nonlinearity is softening, with the oscillation amplitude decreasing as the frequency grows. Softening nonlinearity has also been found within certain parameter ranges in finite-element numerical calculations for vibrating c-c beams [25].

Our results for stationary oscillations in the two-oscillator model show that, even for relatively large values of the Duffing coefficient in the higher harmonic oscillator (up to ten times as large as for the main mode; see Methods), no qualitative changes are observed in the response of the main mode oscillator, as long as the driving force amplitude remains moderate. On the other hand, when the driving force grows, the resonance curve near the internal resonance gap can become strongly distorted.

**Fig 6:**
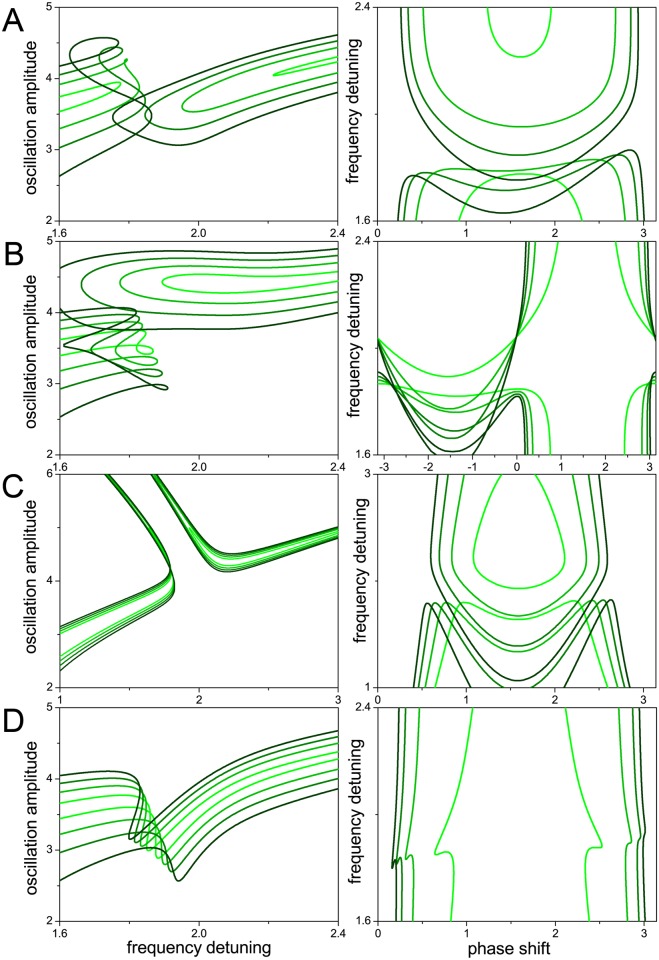
Internal resonance with a nonlinear higher harmonic mode: effect of the driving force. All panels show the zone of the internal resonance gap, with curves for larger force amplitudes plotted with increasingly darker shades of green (see parameters in Table 3). Differences in stability are disregarded. Rows A to D respectively correspond to the coupling forces considered in Figs 2A and 2B and 3A and 3B.

This effect is illustrated in Fig 6, where we present close-ups of the gap for different choices of the parameters, and increasing values of the driving force amplitude (light- to dark-green curves). The higher harmonic cubic nonlinearity is softening. Successive rows correspond to cases with the same coupling forces as in Figs 2 and 3. We see that, as the driving force is increased, the resonance curves may bend and develop foldings that, within certain frequency intervals, imply the appearance of several new possible values for the amplitude. At the same time, in rows A to C, the two branches at each side of the gap approach each other, making the gap narrower. However, whereas in A and B they become mutually superposed for sufficiently large driving forces, they fail to connect with each other (cf. Fig 5B). The gap in the frequency detuning has disappeared, but there is no continuity of solutions across those frequencies. Note additionally that, for the higher harmonic nonlinearity considered in these cases, there is generally no frequency stabilization by internal resonance.

## Methods

### Mathematical Formulation of the Two-Oscillator Model

In the following, we describe the mathematical tools used to obtain the results presented in Figs 1 to 6. As advanced in the preceding section, our model consists of two coupled Duffing oscillators [18]. Emphasizing the fact that they represent two oscillation modes whose frequency ratio is close to 1:3, we denote their coordinates by *x*_1_(*t*) and *x*_3_(*t*), respectively. The equation of motion for the main mode oscillator is

$${\ddot{x}}_{1}+\omega_{1}^{2}x_{1}=\epsilon \left[-2\mu_{1}{\dot{x}}_{1}+\gamma_{1}x_{1}^{3}+\beta_{1}x_{1}^{2}x_{3}+\alpha_{1}x_{1}x_{3}^{2}+\rho_{1}x_{3}^{3}+f_{1}\cos\Omega t\right],$$ (1)

and, for the higher harmonic oscillator,

$${\ddot{x}}_{3}+\omega_{3}^{2}x_{3}=\epsilon \left[-2\mu_{3}{\dot{x}}_{3}+\gamma_{3}x_{3}^{3}+\rho_{3}x_{3}^{2}x_{1}+\alpha_{3}x_{3}x_{1}^{2}+\beta_{3}x_{1}^{3}+f_{3}\cos\left(\Omega t+\theta_{3}\right)\right],$$ (2)

where *ω*_1_ and *ω*_3_ are the corresponding natural frequencies, and *μ*_1_ and *μ*_3_ are the damping coefficients. The coefficients *α*_*i*_, *β*_*i*_, and *γ*_*i*_ (*i* = 1, 3) weight the different cubic forces acting on the oscillators. In particular, *γ*_1_ and *γ*_3_ give the Duffing cubic coefficients, specific to each oscillator. The driving force, which is assumed to act on the two oscillators with amplitudes *f*_1_ and *f*_3_, has frequency *Ω* and appears with a generic phase shift *θ*_3_ is the equation for *x*_3_. Note that all these forces, as well as damping, are normalized by the effective masses of the oscillators. Generally, the coupling forces appearing in Eqs (1) and (2) cannot be derived from an interaction potential and, therefore, are not associated with a conservation law for the mechanical energy. An interaction potential does exist, however, when the coefficients of the coupling terms satisfy the identities *β*_1_/3*β*_3_ = *α*_1_/*α*_3_ = 3*ρ*_1_/*ρ*_3_.

The prefactor *ϵ* in the right-hand side of Eqs (1) and (2) is formally introduced as a small parameter, meaning that all the forces multiplied by *ϵ* are assumed to be small as compared with the linear elastic force in the left-hand side. This assumption is made to apply the multiple-scale approximation to Eqs (1) and (2), as follows [15, 24].

The solutions to Eqs (1) and (2) are expected to depend on time through two different scales. On one side, we have relatively fast oscillations with frequencies of the same order as *ω*_1_, *ω*_3_, and *Ω*. On the other, the oscillation amplitudes and phases change more slowly, allowing for many oscillations to take place within their typical evolution scales. This scale separation is implemented, to the lowest approximation order, by proposing solutions of the form

$$\begin{array}{c} x_{1}\left(\tau_{0},\tau_{1}\right)=A_{1}\left(\tau_{1}\right)\cos\left[\omega_{1}\tau_{0}+\varphi_{1}\left(\tau_{1}\right)\right],\\ x_{3}\left(\tau_{0},\tau_{1}\right)=A_{3}\left(\tau_{1}\right)\cos\left[\omega_{3}\tau_{0}+\varphi_{3}\left(\tau_{1}\right)\right], \end{array}$$ (3)

where the fast variable *τ*_0_ ≡ *t* coincides with the ordinary time *t*, and the slow variable is *τ*_1_ = *ϵt*. The amplitudes *A*_*i*_ and the phases *φ*_*i*_ (*i* = 1, 3) depend on *τ*_1_ only. Moreover, we assume that the frequencies involved in the equations of motion mutually differ by quantities of order *ϵ*, namely

$$\begin{array}{c} \Omega =\omega_{1}+\epsilon \sigma_{1},\\ \omega_{3}=3\omega_{1}+\epsilon \sigma_{3}. \end{array}$$ (4)

Thus, *σ*_1_ and *σ*_3_ measure the detuning between the driving force and the higher harmonic oscillator with respect to the natural frequency of the main mode.

The multiple-scale approximation proceeds by expanding the equations of motion in powers of *ϵ*. Equations for the amplitudes and phases are found by requiring that secular contributions to the fast oscillations vanish [15]. To the first significant order, this procedure yields

$$\begin{array}{c} \omega_{1}A_{1}^{\prime }=-\mu_{1}\omega_{1}A_{1}+\frac{1}{8}\beta_{1}A_{1}^{2}A_{3}\sin\phi_{3}+\frac{1}{2}f_{1}\sin\phi_{1},\\ \omega_{1}A_{1}\varphi_{1}^{\prime }=-\frac{1}{8}\beta_{1}A_{1}^{2}A_{3}\cos\phi_{3}-\frac{1}{4}\alpha_{1}A_{1}A_{3}^{2}-\frac{3}{8}\gamma_{1}A_{1}^{3}+4f_{1}\cos\phi_{1},\\ \omega_{3}A_{3}^{\prime }=-\mu_{3}\omega_{3}A_{3}-\frac{1}{8}\beta_{3}A_{1}^{3}\sin\phi_{3},\\ \omega_{3}A_{3}\varphi_{3}^{\prime }=-\frac{1}{8}\beta_{3}A_{1}^{3}\cos\phi_{3}-\frac{1}{4}\alpha_{3}A_{1}^{2}A_{3}-\frac{3}{8}\gamma_{3}A_{3}^{3}, \end{array}$$ (5)

where the primes are derivatives with respect to the slow variable *τ*_1_. For convenience, we have introduced *ϕ*_1_ = *σ*_1_ *τ*_1_ − *φ*_1_ and *ϕ*_3_ = *σ*_3_ *τ*_1_ + *φ*_3_ − 3*φ*_1_ which are the phase shifts of the driving force and the higher harmonic oscillator with respect to the main mode oscillator. Note that the terms with coefficients *ρ*_1_ and *ρ*_3_, as well as the force on the higher harmonic oscillator, do not contribute to the dynamics at this approximation order.

The explicit form of $\varphi_{1}^{\prime }$ in Eq (5) depends on the working setup being considered. In the open-loop configuration the driving force frequency is fixed, and therefore *σ*_1_ is a constant, which implies $\varphi_{1}^{\prime }=\sigma_{1}-\phi_{1}^{\prime }$. In the closed-loop configuration, on the other hand, the phase shift between the force and the main mode oscillator is fixed, $\phi_{1}^{\prime }=0$, and the force frequency changes. Therefore, $\varphi_{1}^{\prime }=\sigma_{1}+\tau_{1}\sigma_{1}^{\prime }$. As for $\varphi_{3}^{\prime }$, since *σ*_3_ is constant in both configurations, we have $\varphi_{3}^{\prime }=3\varphi_{1}^{\prime }-\sigma_{3}+\phi_{3}^{\prime }$. This yields $\varphi_{3}^{\prime }=3\sigma_{1}-\sigma_{3}+\phi_{3}^{\prime }-3\phi_{1}^{\prime }$ and $\varphi_{3}^{\prime }=3\sigma_{1}-\sigma_{3}+\phi_{3}^{\prime }-3\sigma_{1}^{\prime }\tau_{1}$ for the open- and close-loop configuration, respectively. Hence, in addition to the unknowns *A*_1_, *A*_3_, and *ϕ*_3_, Eq (5) are differential equations for *ϕ*_1_ in the open-loop configuration and for *σ*_1_ in the closed-loop configuration. This difference must be taken into account, for instance, when analyzing the stability of the stationary solutions to the equations.

On the other hand, as mentioned in the preceding section, the stationary solutions are the same in both configurations. Respectively replacing $\varphi_{1}^{\prime }$ and $\varphi_{3}^{\prime }$ by *σ*_1_ and 3*σ*_3_ − *σ*_1_, and taking $A_{1}^{\prime }=A_{3}^{\prime }=0,$ we obtain the following algebraic equations for the stationary solutions:

$$\begin{array}{c} 8\mu_{1}\omega_{1}A_{1}-\beta_{1}A_{1}^{2}A_{3}\sin\phi_{3}-4f_{1}\sin\phi_{1}=0,\\ 8\omega_{1}A_{1}\sigma_{1}+\beta_{1}A_{1}^{2}A_{3}\cos\phi_{3}+2\alpha_{1}A_{1}A_{3}^{2}+3\gamma_{1}A_{1}^{3}+4f_{1}\cos\phi_{1}=0,\\ 8\mu_{3}\omega_{3}A_{3}+\beta_{3}A_{1}^{3}\sin\phi_{3}=0,\\ 8\omega_{3}A_{3}\left(3\sigma_{1}-\sigma_{3}\right)+\beta_{3}A_{1}^{3}\cos\phi_{3}+2\alpha_{3}A_{1}^{2}A_{3}+3\gamma_{3}A_{3}^{3}=0. \end{array}$$ (6)

These are the equations which were solved to get the results presented in Figs 1 to 6. Solutions were found numerically, using a standard multidimensional Newton-Raphson algorithm [26]. Tables 1 to 3 show the parameters used in each figure.

**Table 2:** Parameters used in Eq (6) to obtain the results plotted in Fig 5.

Fig	5A	5B	5C	5C
*β*_1_	0	0.15	0	0
*α*_1_	0	0	1	−1
*f*_1_	1, 2, 5, 10	1, 2, 3, 4, 5	1, 2, 5, 10	1, 2, 5, 10

The remaining parameters are *ω*_1_ = 1; *ω*_3_ = 9; *σ*_3_ = 6; *μ*_1_ = *μ*_3_ = 0.1; *γ*_1_ = −0.33; *γ*_3_ = 0; *β*_3_ = 0.5; *α*_3_ = 0.

**Table 3:** Parameters used in Eq (6) to obtain the results plotted in Fig 6.

Fig	6A	6B	6C	6D
*β*_1_	0.5	−0.5	0	0
*α*_1_	0	0	1	−1
*f*_1_	1, 2, 3, 4	1, 2, 3, 4	1, 1.2, 1.4, 1.6	1, 2, 3, 4

The remaining parameters are *ω*_1_ = 1; *ω*_3_ = 9; *σ*_3_ = 6; *μ*_1_ = *μ*_3_ = 0.1; *γ*_1_ = −0.33; *γ*_3_ = 3; *β*_3_ = 0.5; *α*_3_ = 0.

### Backbone approximation and estimation of the frequency gap

The backbone curve is an approximation for the resonance peak of the Duffing oscillator, which schematically reproduces its profile at a significantly lower cost in mathematical complexity [15]. The left panel of Fig 1 shows that the backbone curve for a single forced Duffing oscillation, whose equation is

$$A_{1}=\sqrt{-\frac{8\omega_{1}}{3\gamma_{1}}\sigma_{1}},$$ (7)

gives a quantitatively satisfactory description of the effect of nonlinearity on the resonance peak. In this case, the approximation is obtained by assuming that damping and the driving force are both negligible as compared with the restoring (linear and cubic) forces.

For our two coupled oscillators, a backbone-like approximation can be obtained in the special case where the cubic restoring force of the higher harmonic oscillator is absent, *γ*_3_ = 0. In this case, the two last of Eq (6) are linear in *A*_3_, which makes it possible to immediately obtain *A*_3_ as a function of *A*_1_:

$$A_{3}=\frac{|\beta_{3}|A_{1}^{3}}{2\sqrt{\alpha_{3}^{2}A_{1}^{4}+8\omega_{3}\alpha_{3}\Delta A_{1}^{2}+16\omega_{3}^{2}\left(\Delta^{2}+\mu_{3}^{2}\right)}},$$ (8)

with Δ = 3*σ*_1_ − *σ*_3_. From the same equations, we find

$$A_{3}\cos\phi_{3}=-\frac{\beta_{3}A_{1}^{3}\left(2\omega_{3}\Delta +\frac{1}{2}\alpha_{3}A_{1}^{2}\right)}{\alpha_{3}^{2}A_{1}^{4}+8\omega_{3}\alpha_{3}\Delta A_{1}^{2}+16\omega_{3}^{2}\left(\Delta^{2}+\mu_{3}^{2}\right)}.$$ (9)

Replacing Eqs (8) and (9) into the second of Eq (6) and taking the limit *f*_1_ → 0, a higher-order equation for *A*_1_ is obtained. An approximated solution to this equation can in turn be found under the assumption that the coupling forces acting on the main mode oscillator are small as compared with its restoring forces, yielding

$$A_{1}^{2}=A_{*}^{2}-\frac{\beta_{3}}{3\gamma_{1}}\frac{\frac{1}{2}\alpha_{1}\beta_{3}A_{*}^{6}-\beta_{1}\left(2\omega_{3}\Delta +\frac{1}{2}\alpha_{3}A_{*}^{2}\right)A_{*}^{4}}{\alpha_{3}^{2}A_{*}^{4}+8\omega_{3}\alpha_{3}\Delta A_{*}^{2}+16\omega_{3}^{2}\left(\Delta^{2}+\mu_{3}^{2}\right)},$$ (10)

where $A_{*}=\sqrt{-8\omega_{1}\sigma_{1}/3\gamma_{1}}$ is nothing but the backbone curve for the uncoupled main mode, Eq (7). The graphs of *A*_1_ as a function of *σ*_1_, as given by Eq (10), are the backbone curves plotted in Figs 1 to 5.

Finally, taking these results as the lowest order approximation to the solution of the second to fourth of Eq (6), we replace them in the first equation and solve for sin *ϕ*_1_, getting

$$\sin\phi_{1}=\frac{2A_{1}}{f_{1}}\left[\omega_{1}\mu_{1}+\frac{\frac{1}{4}\beta_{1}\beta_{3}\omega_{3}\mu_{3}A_{1}^{4}}{\alpha_{3}^{2}A_{1}^{4}+8\omega_{3}\alpha_{3}\Delta A_{1}^{2}+16\omega_{3}^{2}\left(\Delta^{2}+\mu_{3}^{2}\right)}\right].$$ (11)

The frequency gap caused by internal resonance occurs when this equation does not provide a (real) solution for *ϕ*_1_, i.e. when

$$\frac{2A_{1}}{f_{1}}\left|\omega_{1}\mu_{1}+\frac{\frac{1}{4}\beta_{1}\beta_{3}\omega_{3}\mu_{3}A_{1}^{4}}{\alpha_{3}^{2}A_{1}^{4}+8\omega_{3}\alpha_{3}\Delta A_{1}^{2}+16\omega_{3}^{2}\left(\Delta^{2}+\mu_{3}^{2}\right)}\right|>1.$$ (12)

This inequality is satisfied inside the gap, and does not hold outside.

## Conclusion

In this paper, we have examined the stationary motion of a system of two coupled nonlinear oscillators, driven by a harmonic force, where restoring and coupling forces are cubic functions of the oscillation amplitudes. The system is conceived as a model for the interaction between the main oscillation mode and a higher harmonic mode of a vibrating clamped-clamped elastic beam. When the ratio between the natural frequencies of the two modes is close to 1:3, cubic nonlinearity is most effective in coupling the respective oscillations, which synchronize with each other, and internal resonance thus takes place. Internal resonance has recently been proposed as a mechanism to neutralize the amplitude-frequency interdependence in micromechanical oscillators [16], which threatens their possible application as pacemakers in the design of time-keeping miniaturized devices.

Our emphasis has been put in illustrating the diversity of behavior that various choices of the parameters controlling the coupling between modes bring about in the stationary response of the main mode oscillation, in particular, in the vicinity of internal resonance. Although –because of the multiplicity of those parameters– a systematic exploration of parameter space is not possible, the selection of cases presented in the results summarizes the main effects of different forms of coupling that a more extensive analysis shows to occur. We have also paid attention to the effects of changing the strength of the driving force, which is a key control parameter in applications of vibrating c-c beams. Our presentation has been limited to results on the main mode oscillation, which is most readily accessible to experimental observation –directly through electrical measurements. The mathematical formulation presented in the Methods section, however, provides the tools for studying the higher harmonic dynamics as well.

It is important to point out that our approach to the dynamics of c-c beams has been based on deterministic equations of motion, while in the Introduction we mentioned that in some applications –specifically, those related to microtechnology– noise may play an important role in establishing the working conditions of the oscillator [12–14]. The effect of stochastic forces can generically be predicted taking into account that internal resonance is the consequence of the occurrence of direct and inverse saddle-node bifurcations [27] in the dynamical system under study. This brings about the possibility that its response to noise has the form of one or more well-studied phenomena in stochastic nonlinear systems, ranging from noise-induced transitions to bifurcation postponement, and stochastic phase locking [28]. Being a system of forced coupled oscillators, noise may also prompt the occurrence of stochastic resonance with the external force and/or between the two interacting modes [29]. Nonetheless, experiments with actual micromechanical oscillators have revealed robustness against noise under standard conditions [16].

The appearance of a gap in the Duffing resonance curve, separating the possible stationary oscillations of the system into two disconnected branches, is the most evident signature of the occurrence of internal resonance. The gap has been directly observed in clamped-clamped micromechanical oscillators vibrating in closed-loop configurations [17, 24], and is also responsible for the behaviour reported in open-loop experiments with both micro- [16] and nanomechanical oscillators [30, 31]. Similarly, the appearance of an “island” in the response of a single forced oscillator has been predicted for subharmonic resonances, when the driving force frequency is close to one third the natural oscillation frequency [15]. Very recently, analogous effects have been found in related systems such as vibrating electroelastic crystals [32] and velocity-coupled nonlinear oscillators [33].

Application of the two-oscillator model to a quantitative fitting of experimental or numerical results on c-c beam vibration, which we have not attempted here, makes it necessary to estimate the model parameters for each particular instance. This estimation would in turn require detailed information on the set-up under consideration –for example, in an experiment, the design of circuits that transduce mechanical motion into electric signals. Moreover, in some specific experiments, such as those involving micromechanical oscillators, there are huge quantitative differences between the involved forces –e.g., a ratio as large as 10^5^ between the elastic force and damping [16]– which result into parameter values which differ from each other by several orders of magnitude. In such situation, dealing with the equations derived from the mathematical approach demands more sophisticated numerical algorithms than those used in our calculations.

On the other hand, several of the results for our two-oscillator system are in qualitative agreement with specific forms of behavior observed in clamped-clamped beams. For instance, the closure of the internal resonance gap upon increasing the driving force (cf. Fig 5B) has been reported from experiments on micromechanical oscillators [24]. The downward bending of the Duffing resonance curve near internal resonance (cf. Figs 3B and 4B) has also been obtained from finite-element computation of c-c beam nonlinear vibrations. The profile of the resonance curve near the gap in the cases considered in Fig 5C is the same as successively reported since long ago, both experimentally and theoretically, for different types of vibrating structures [19, 34, 35]. These similarities point to the versatility of the two-oscillator model as an approximate description of mode interaction in this kind of system.
